# Evolutionary Repurposing of Cytokinin Signaling in Plant Development and Symbiosis

**DOI:** 10.3390/plants15091370

**Published:** 2026-04-30

**Authors:** Shiqi Zhang, Yanping Jiang, Jianing Fang, Tao Wang

**Affiliations:** 1 College of Life Sciences, Zhejiang Normal University, Jinhua 321004, China; 2 Xianghu Laboratory, Hangzhou 311231, China

**Keywords:** cytokinin, evolution, transport, hormone crosstalk, arbuscular mycorrhiza, nodulation, symbiosis

## Abstract

Cytokinin (CK) is a central regulator of plant development, yet its roles cannot be understood fully without considering how CK signaling was assembled during evolution and redeployed in different physiological contexts. In this review, we examine how prokaryotic two-component modules were elaborated into the land–plant CK system and how this system now integrates biosynthesis, transport, receptor selectivity, and feedback control to shape developmental and symbiotic outcomes. We argue that three recurring interpretive dimensions are especially useful for organizing current evidence: compartmentalized CK pools, context-dependent decoding of local CK availability, and the coupling of local CK responses to whole-plant nutrient status. These dimensions help organize current observations on why CK effects in arbuscular mycorrhiza (AM) are often conditional and readout-dependent, whereas evidence from legume–rhizobium symbiosis supports a more direct role for CK in cortical competence, nodule organogenesis, and autoregulation of nodulation. Rather than treating CK as a generic positive regulator of symbiosis, we propose that it functions as a spatially partitioned and nutritionally gated integrator whose outputs depend on cell type, developmental stage, transport route, and resource context. We conclude by highlighting key mechanistic gaps—particularly in transporter-resolved CK partitioning and systemic integration—and by outlining experimentally testable priorities for translating CK biology into crop improvement.

## 1. Introduction

Cytokinins (CKs), a class of N6-substituted adenine derivatives, were initially discovered and named for their remarkable ability to promote plant cell division [1]. First identified in the 1950s from coconut milk, herring sperm DNA, and maize kernels, CKs are now recognized as multifunctional hormones that regulate diverse developmental and physiological processes, including embryogenesis, meristem activity, organ development, senescence, and source–sink relationships [2,3].

Here, we first revisit how the cytokinin toolkit was assembled from ancestral signaling components into a spatially and metabolically diversified regulatory system. We then examine how modern CK biology in angiosperms depends on local biosynthesis, transport-defined partitioning, receptor selectivity, and hormonal crosstalk. Finally, we evaluate how these modules are redeployed in plant–microbe interactions, with particular emphasis on the contrast between the conditional, context-dependent roles of CK in arbuscular mycorrhiza and the better-supported organogenic functions of CK in legume nodulation. Throughout, we distinguish between established mechanisms, evidence-based working models, and forward-looking hypotheses.

Here, we use the term evolutionary repurposing to refer to the co-option and redeployment of pre-existing CK signaling modules in new developmental, physiological, or symbiotic contexts, rather than to the invention of entirely new signaling logic. To keep the argument proportional to the available evidence, we distinguish throughout between (i) mechanisms that are genetically and cell-biologically well supported, (ii) integrative models that are strongly suggested but not yet quantitatively closed, and (iii) forward-looking hypotheses proposed to organize future work. Accordingly, terms such as threshold, gradient, resource satiation, and systemic budgeting are used here as explicit working constructs unless direct causal evidence is stated. In most cases, the relevant variable is not bulk cytokinin abundance alone, but the effective signal perceived by a defined cell population after local synthesis, transport, turnover, receptor selectivity, and developmental context are taken into account [4,5].

As an organizing framework, this review emphasizes three recurring interpretive dimensions in the literature: compartmentalized CK pools, context-dependent decoding of CK availability, and the coupling of local CK responses to whole-plant resource status. We use these dimensions to organize diverse observations, while recognizing that they are not equally resolved mechanistically across systems and should not be read as a single universal explanatory model.

## 2. Evolutionary Assembly of Cytokinin Signaling

The evolutionary history of the CK system illustrates how ancestral signaling modules can be repeatedly repurposed during plant diversification. Gene co-option, duplication, neofunctionalization, and loss have contributed to the increasing regulatory complexity of CK metabolism and signaling, broadly paralleling the expansion of plant morphological and ecological diversity. Reconstructing this history does not yield a single linear trajectory, but it does provide a useful comparative framework for understanding why CK outputs differ among lineages and biological contexts.

Editorial note on evolutionary inference: Reconstructing deep-time cytokinin signaling evolution is inherently uncertain and frequently lineage-specific. Apparent ‘gains’ of pathway components may reflect differential retention after duplication, hidden homology, or annotation bias; conversely, losses and functional rewiring can obscure ancestral states. Throughout this review, statements about the timing and direction of innovations should therefore be interpreted as comparative, best-supported scenarios rather than strictly linear progressions, and alternative trajectories (convergent recruitment, sub-functionalization, and loss) should be considered where evidence is limited.

Accordingly, references in the following sections to “ancestral”, “derived”, or “expanded” cytokinin modules should be read as comparative shorthand rather than as implying a linear progression from simple to superior systems. In several cases, the most defensible conclusion is not that a pathway was uniformly gained, but that distinct lineages retained, elaborated, or repurposed different subsets of an older signaling toolkit [3,6,7].

### 2.1. The Ancient Origins of the Plant Two-Component Signaling System

The cytokinin signaling pathway in plants is a specialized multi-step phosphorelay (MSP) system derived from the prokaryotic two-component system (TCS) [8]. A canonical bacterial TCS comprises a membrane-bound histidine kinase (HK) that perceives environmental signals and a response regulator (RR) that mediates cellular outputs [9].

The plant MSP system likely originated through ancient acquisition and repurposing of prokaryote-like two-component signaling modules, potentially via endosymbiotic or horizontal gene transfer followed by extensive neofunctionalization [10]. This scenario is consistent with comparative genomic data, but alternative evolutionary histories remain plausible, and direct deep-time reconstruction is necessarily inferential. The key point for this review is therefore not a single linear origin story, but the repeated biological reuse of ancestral phosphorelay logic in increasingly compartmentalized and developmentally sophisticated plant contexts.

Plants subsequently elaborated this ancestral module into a more complex His-Asp-His-Asp phosphorelay. This system features hybrid HKs containing both HK and RR domains, mobile histidine phosphotransfer proteins (HPts) that shuttle between cytoplasm and nucleus, and type-A and type-B RRs [8]. The HPt proteins act as critical signal shuttles, increasing pathway flexibility and creating nodes for signal integration [11]. This “evolutionary tinkering”—repurposing a prokaryotic sensory system for endogenous developmental control—was a key innovation enabling the transition to multicellularity in plants.

### 2.2. Assembly of the Cytokinin Toolkit

The full CK system depends on coordinated gene families involved in biosynthesis, activation, degradation, perception, and signal transduction. These components did not emerge simultaneously, nor did they diversify along a strictly linear route. Instead, current comparative analyses support a stepwise assembly in which different modules expanded at different times during streptophyte and land-plant evolution, broadly tracking major transitions in plant body plan complexity and ecological adaptation [6].

Comparative caveat: current reconstructions of cytokinin toolbox assembly remain sensitive to phylogenomic sampling, genome annotation quality, and the fact that sequence homology does not guarantee conserved biochemical or developmental function. Throughout this section, inferred gains, losses, or expansions should therefore be read as the best-supported comparative scenario rather than as a fully closed evolutionary trajectory.

### 2.3. Biosynthesis and Activation: Divergent Routes, Convergent Function for IPT and LOG Gene Families

Isopentenyltransferases (IPTs) catalyze the rate-limiting step in the de novo biosynthesis of cytokinins: the transfer of an isopentenyl group to an adenine nucleotide, forming the crucial side chain [12,13]. Its evolutionary history is particularly complex and intriguing. Phylogenetic analyses reveal that plant IPT genes responsible for free cytokinin synthesis are surprisingly more closely related to the bacterial miaA gene, which modifies tRNA and does not produce free cytokinins, rather than to bacterial adenosine-type IPT genes that do synthesize free cytokinins [12]. This remarkable finding indicates that the ability to synthesize free cytokinins arose independently on two separate occasions—in plants and in some bacteria—thereby demonstrating a striking case of convergent evolution.

Within the plant kingdom, the IPT gene family diversified into several clades that differ in substrate preference, subcellular localization, and phylogenetic distribution, indicating that biosynthetic capacity expanded through repeated specialization rather than through a single uniform innovation.

Class I *tRNA-IPTs*: Regarded as direct descendants of the bacterial miaA gene, they are retained across all plant lineages, from algae to angiosperms. While cis-Zeatin (*c*Z) and its derivatives produced by these tRNA-IPTs are typically regarded as having low bioactivity, they nonetheless function in specific developmental contexts and stress adaptation [6].

Class II *tRNA-IPTs*: Of probable eukaryotic origin, these genes have been reported in selected algal and vascular plant lineages, although their precise phylogenetic distribution and ancestry remain incompletely resolved.

Adenosine phosphate-*IPTs* (*AP-IPTs*): These enzymes are the major source of the highly active CKs trans-zeatin (*t*Z) and isopentenyladenine (iP) in land plants. Current phylogenetic models are consistent with their origin from an ancestral *tRNA-IPT*-related lineage followed by functional divergence in early land plants, after which *AP-IPTs* expanded substantially in angiosperms.

While isopentenyltransferases (IPTs) catalyze the committed step in CK biosynthesis, bioactive CK production also requires removal of the ribose-5′-monophosphate moiety from cytokinin nucleotides. This reaction is catalyzed by LONELY GUY (LOG) phosphoribohydrolases, which convert inactive nucleotides into active free bases [14,15,16]. In contrast to the complex evolutionary history inferred for IPT genes, LOG homologs occur in unicellular algae and across land-plant lineages, consistent with an ancient origin of CK activation capacity [17,18]. Subsequent lineage-specific expansion and subfunctionalization of LOG genes likely increased the spatiotemporal precision of local CK activation. In addition, emerging evidence points to non-canonical or LOG-independent contributions to CK production in some contexts, suggesting that the biosynthetic network may be more flexible than the canonical IPT-LOG model alone implies [6].

### 2.4. Homeostatic Control: The Emergence and Expansion of the CKX Degradation Pathway

To counterbalance the activity of CKs generated by the IPT-LOG module, cytokinin oxidase/dehydrogenase (CKX) enzymes act as major homeostatic regulators by irreversibly cleaving the N6 side chain of active CKs such as iP and *t*Z [19]. *CKX* genes form small multigene families whose copy number varies among species; for example, *Arabidopsis thaliana* has seven *CKX* genes, *Oryza sativa* eleven, and *Zea mays* thirteen [20]. Much of this expansion appears to have occurred through segmental duplication, creating opportunities for subfunctionalization. Indeed, *CKX* isoforms differ in subcellular localization, substrate preference, and spatiotemporal expression, indicating that they are not simply redundant degradative enzymes but components of a distributed regulatory system that fine-tunes CK levels in specific tissues and developmental contexts [19,20].

Beyond developmental tuning, *CKX* is increasingly viewed as a tunable resistor for balancing productivity and stress resilience in crops. In indica rice, CRISPR/Cas9-generated cytokinin oxidase2-deficient mutants (*OsCKX2*) accumulate cytokinin and display improved panicle branching and grain yield, while also enhancing drought tolerance via reduced transpiration, improved membrane/chloroplast integrity and strengthened antioxidant protection [21].

### 2.5. Perception and Signaling Modules

Histidine kinase (HK) receptors are the primary sensors of CK signals [8]. A major evolutionary step was the acquisition of CHASE-domain-containing HK receptors able to perceive adenine-derived ligands with increasing specificity. In extant land plants, receptor families diversified further, but this diversification should not be interpreted as a simple linear progression toward higher complexity. Rather, different lineages retained and elaborated receptor repertoires in ways that likely reflect species-specific developmental and ecological demands.

A quantitative evolutionary view also requires careful consideration of receptor–ligand selectivity. Available biochemical and structural studies show that plant CK receptors differ in affinity for distinct CK species, but the extent, physiological significance, and evolutionary origin of this selectivity are still being refined. Recent preprint-level evidence is intriguing in proposing mechanisms of CK and receptor binding diversity, yet it is crucial to note that these findings await formal peer review. The field still requires broader comparative validation across lineages and receptor classes before strong generalizations can be made regarding the structural basis of evolutionary divergence in ligand preference.

At the same time, receptor selectivity should not be overinterpreted as a fixed biochemical code detached from tissue context. Apparent ligand preference can be reshaped by receptor abundance, subcellular localization, access to local cytokinin pools, and the dynamics of feedback attenuation through Type-A RRs and CKX activity. For this reason, biochemical affinity, structural inference, and developmental output should be considered complementary rather than interchangeable layers of evidence [22,23].

In angiosperms, the cytokinin receptor gene family underwent further diversification, giving rise to three major subfamilies in *Arabidopsis thaliana*: *AHK2*, *AHK3*, and *CRE1/AHK4* [24]. These receptors exhibit both functional redundancy and specificity [7]. For instance, in the *Arabidopsis* root, *CRE1/AHK4* plays a predominant role, while *AHK2* and *AHK3* provide significant redundant functions [25]. Although the triple mutant (*CRE1 AHK2 AHK3*) is viable, it is completely unresponsive to cytokinin. This finding conclusively demonstrates the central and non-redundant role of these three receptors in cytokinin perception [25].

Response regulators (RRs) act as the final effectors of the signaling pathway. They have also undergone functional specialization during evolution: Type-B response regulators (Type-B RRs) contain a DNA-binding domain and function as transcriptional activators that initiate the expression of downstream target genes [26]. In contrast, Type-A response regulators (Type-A RRs) are themselves target genes of Type-B RRs and are rapidly induced by cytokinin. However, they lack a DNA-binding domain and instead act as negative feedback regulators by competing with Type-B RRs for phosphorylation from the HPt protein [8]. The “one positive, one negative” regulatory pair formed by Type-A and Type-B RRs constitutes a canonical and efficient negative feedback loop, which ensures the transient yet robust nature of cytokinin signaling. Phylogenetic analyses reveal that both Type-A and Type-B RRs are present in all land plants, indicating that this core regulatory architecture was already established at the dawn of plant terrestrialization [26]. In addition, a smaller subfamily of Type-C response regulators (Type-C RRs) exists, whose functions are not yet fully understood [26]. The major evolutionary adaptations discussed in this section, together with their functional significance and comparative interpretation, are summarized in Table 1.

### 2.6. A Glimpse into Ancestral Function: Cytokinin Signaling in Bryophytes (Physcomitrella, Marchantia)

As early-diverging land plants, *Physcomitrium* (*Physcomitrella*) *patens* and *Marchantia polymorpha* provide an informative, although not complete, window into ancestral CK functions. Their value lies less in representing a direct evolutionary snapshot than in revealing which core features of CK signaling were already established near the base of land–plant diversification.

In *Physcomitrella patens*, cytokinin acts as the core signaling molecule that regulates a pivotal developmental transition in its life cycle: the shift from filamentous protonemal growth to the formation of leafy gametophores [29]. Studies of *CKX*-overexpressing mutants (with reduced CK levels) and CK-overproducing mutants have revealed that the extracellular iP and its nucleoside iPR, which predominantly accumulate in the culture medium, are the primary active forms responsible for inducing this process [29]. This finding suggests that in early land plants, cytokinins likely regulated major developmental program switches primarily as extracellular signals. This may represent a more ancestral mode of action, preceding the evolution of the sophisticated intracellular and long-distance transport systems seen in higher plants.

In *Marchantia polymorpha*, research has confirmed that it possesses a complete and conserved cytokinin signaling pathway, including homologs of *CKX*, Type-A RR (*MpRRA*), and Type-B RR (*MpRRB*) [1]. Precise manipulation of this pathway via gene editing reveals that cytokinin is essential for the formation of multiple organs on the thallus; it promotes the development of asexual reproductive gemma cups and stomata, while suppressing rhizoid growth [7]. Crucially, the negative feedback loop in which Type-B RRs activate the expression of Type-A RRs—which in turn regulate the activity of Type-B RRs—is conserved in Marchantia [30].

Taken together, bryophyte studies support the view that CK had already become an important regulator of cell proliferation, organ formation, and developmental transitions early in land-plant evolution. However, these data should not be read as evidence that the full regulatory logic of angiosperm CK networks was already present. Instead, bryophytes most likely preserve a subset of ancestral functions onto which later layers of tissue specificity, long-distance transport, and hormone crosstalk were progressively added.

Evidence status summary for bryophytes: Support is strongest for conserved roles in cell proliferation, organ initiation, and receptor-mediated signaling logic. Much weaker support exists for directly extrapolating bryophyte cytokinin functions to the later emergence of vascular developmental complexity or symbiotic specializations in seed plants.

### 2.7. The Modern Cytokinin Regulatory Framework in Angiosperms

The transition from the relatively simple CK network observed in bryophytes to the highly elaborated system of angiosperms reflects repeated gains in spatial control, feedback tuning, metabolic diversification, and inter-organ communication. Rather than implying a teleological march toward complexity, this pattern is better viewed as lineage-specific expansion of regulatory options that enabled CK signaling to be deployed across more cell types, organs, and environmental contexts.

## 3. The Core Framework for Cytokinin System Establishment: From Perception and Signal Transduction to Transport

### 3.1. Perception and Signal Transduction: A High-Fidelity Phosphorelay Network

Cytokinin signal transduction in angiosperms is a canonical multi-step phosphorelay (MSP) process whose fidelity depends on the sequential transfer of phosphate through a series of signaling proteins [8]. Importantly, this pathway does not merely relay CK perception; its layered architecture also provides a molecular basis for context-dependent decoding by allowing signal strength, duration, and transcriptional output to vary with receptor composition, phosphotransfer competition, and feedback attenuation.

(1) Signal perception: The signal is initiated by the binding of a cytokinin molecule to the CHASE domain of a receptor dimer located on the endoplasmic reticulum or plasma membrane, as exemplified by *AHK4/CRE1* in *Arabidopsis* [31].
(2) Receptor activation and phosphate transfer: Ligand binding induces a conformational change in the receptor, prompting its kinase domain to undergo trans-autophosphorylation, whereby one monomer phosphorylates a conserved histidine (His) residue on the other monomer. Subsequently, the phosphate group is transferred intramolecularly to a conserved aspartate (Asp) residue within its own receiver (Rec) domain [32].
(3) Cytoplasmic–nuclear shuttle: Phosphorylated receptors transfer phosphate groups to histidine phosphotransfer proteins (AHPs/HPts), which maintain a nucleo-cytosolic distribution and mediate phosphotransfer between the cytoplasm and nucleus [33].
(4) Nuclear signal transmission: In the nucleus, phosphorylated HPts transfer the phosphate group to a conserved Asp residue within the receiver domain of Type-B RRs (RRBs), which act as the main transcriptional effectors of the pathway [32,34].
(5) Transcriptional activation: Phosphorylation activates the Type-B RR, enabling it to bind cytokinin-responsive cis-elements that contain a core (A/G)GAT motif in the promoters of downstream target genes and thereby initiate transcription [34].

To ensure that CK signaling remains transient and adjustable rather than constitutively amplified, a negative feedback loop is built into the pathway. Activated Type-B RRs rapidly induce the expression of Type-A RR (RRA) genes. Type-A RR proteins lack a DNA-binding domain, but their receiver-like structure allows them to act as competitive phospho-accepting decoys that divert phosphate flux away from Type-B RRs [8,35]. This competition attenuates signal propagation and helps reset the pathway to its basal state after stimulation. More importantly, such feedback control provides a mechanistic basis for signaling precision: cells do not simply register the presence or absence of CK, but interpret CK inputs according to receptor complement, phosphotransfer dynamics, and feedback strength. In this sense, the MSP pathway is not only a transmission system, but also a decoding system that helps explain why similar CK signals can produce different outputs in different developmental and physiological contexts.

**Figure 1:**
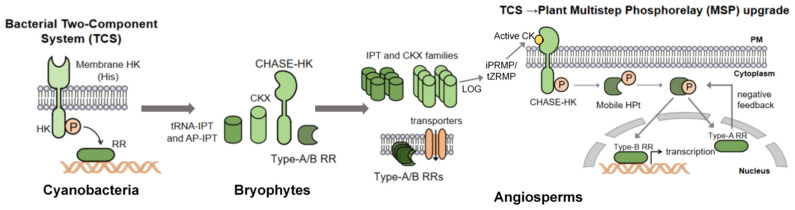
Evolution and elaboration of the cytokinin multi-step phosphorelay (MSP) signaling architecture. The schematic summarizes how an ancestral prokaryotic two-component system (TCS) was progressively co-opted and elaborated into the plant MSP pathway. Solid arrows indicate relationships that are broadly supported by comparative, genetic, and biochemical evidence.

Such decoding, however, depends not only on phosphorelay architecture after perception, but also on how CK availability is spatially partitioned before perception. This makes CK transport a central determinant of where distinct CK pools are formed and which cell populations are exposed to them. A schematic overview of the evolutionary elaboration of this phosphorelay architecture, from ancestral two-component modules to the modern plant MSP system, is provided in Figure 1.

### 3.2. Establishing Spatiotemporal Gradients: The Cytokinin Transport Machinery

The biological effects of cytokinins depend not only on their overall abundance, but also on how distinct CK forms are partitioned across cells, tissues, and transport routes. The establishment and maintenance of these spatiotemporal gradients require a transporter network that controls intracellular compartmentalization, short-distance intercellular movement, and long-distance communication between organs [36]. In this sense, CK transporters do not merely redistribute hormone molecules; together, they generate compartmentalized CK pools, thereby shaping which cells perceive CK, when they perceive it, and how strongly they respond. This transport-defined partitioning becomes especially consequential in symbioses, where epidermal entry, cortical accommodation, and long-distance nutrient signaling must be coordinated across distinct tissues. Four major transporter families have been implicated in these processes.

Purine Permeases (PUPs): PUPs function primarily as influx carriers for cytokinin free bases such as *t*Z and for adenine, thereby importing extracellular cytokinins into cells [37]. The family comprises numerous members with diverse tissue-specific expression patterns. For example, *AtPUP2* in *Arabidopsis* is expressed in vascular phloem tissues and has been proposed to participate in long-distance transport, whereas *AtPUP14* appears to regulate signal perception by removing apoplastic cytokinins and thereby buffering extracellular CK availability [38]. In legumes, *Lotus japonicus LjPUP1* has been identified as a cytokinin efflux transporter implicated in exporting CKs synthesized in root nodules [37]. Mechanistically, transporters of this class matter not simply because they move CKs, but because they can bias where particular CK pools remain apoplastic, become cell-associated, or are redirected into vascularly connected routes. In symbiotic settings, such partitioning is expected to influence which cell layers are exposed to bioactive free-base CKs, even though CK species-specific fluxes in nodules are not yet quantitatively resolved. Conceptually, these examples show that PUP proteins can restrict or redirect local CK availability, helping separate extracellular, intracellular, and vascularly connected CK pools rather than allowing CK to function as a uniform signal background.

Equilibrative Nucleoside Transporters (ENTs): ENTs primarily transport the nucleoside forms of cytokinins, such as *t*ZR. In *Arabidopsis thaliana*, *AtENT3* contributes to the partitioning of cytokinins between the apoplast and symplast in the root. By promoting nucleoside uptake into cells, it restricts their further long-distance transport to the shoot through the xylem [12]. Thus, ENTs help define which CK forms remain available for local use and which enter systemic transport streams, adding a chemical dimension to CK compartmentalization.

ATP-Binding Cassette (ABC transporters): ABC transporters, particularly members of the ABCG subfamily, function mainly as efflux pumps in plant CK transport. *Arabidopsis AtABCG14* is a key regulator of long-distance transport: it is expressed in root pericycle cells and loads root-synthesized *t*Z-type cytokinins into the xylem for efficient shootward movement [39,40]. In legumes, *Medicago truncatula MtABCG56* is a Nod factor-induced cytokinin efflux transporter expressed in root epidermal and cortical cells [36]. Its mechanistic relevance is that it provides a plausible route by which CK pools could be redistributed between infection-competent outer tissues and cortex-associated organogenic domains, thereby helping coordinate epidermal infection progression with cortical cell division rather than allowing both processes to experience the same effective CK environment. Which CK species dominate this flux, and how strongly this redistribution contributes quantitatively to the epidermis-cortex balance under physiological conditions, remain unresolved. Conversely, *MtABCG40* has been identified as a cytokinin influx transporter that negatively regulates nodule number [37]. Taken together, these examples suggest that opposing influx and efflux activities are likely central to generating tissue-specific CK domains rather than a single diffusible CK field. Aza-Guanine Resistance (AZG transporters): AZG1 and AZG2, the most recently identified class of CK transporters in *Arabidopsis*, transport cytokinins and also influence lateral root growth through interaction with the auxin efflux carrier PIN1, particularly under salt stress conditions [41]. Although their symbiotic roles remain unresolved, they further support the view that CK transport cannot be separated from broader developmental and hormonal context.

**Figure 2:**
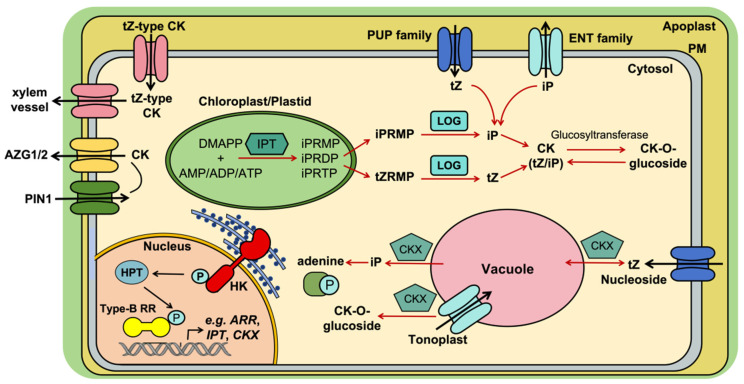
Dedicated transporter families generate compartmentalized cytokinin (CK) pools and enable long-distance coordination of development and symbiosis. The figure summarizes major CK transport routes, the chemical forms preferentially moved by each transporter class, and the developmental or symbiotic contexts in which transport-linked CK partitioning is most likely to matter. Where direct mechanistic validation remains limited, transport-to-phenotype links should be read as working models rather than fixed rules.

Overall, transporter families differ not only in substrate preference and cellular localization, but also in the spatial scale at which they act. Some buffer extracellular CK availability, some partition nucleoside and free-base pools, and others drive directional root-to-shoot or tissue-to-tissue movement. Their combined action therefore provides the most direct mechanistic basis for compartmentalized CK pools, while also helping explain how similar CK molecules can be decoded differently across developmental and symbiotic contexts. In symbiosis, this principle is especially important because transport-based CK partitioning may help uncouple, coordinate, or sequence epidermal infection, cortical organogenesis, and systemic nutrient signaling rather than forcing them into a single uniform hormonal output. Even so, direct species-resolved evidence linking specific transport fluxes to specific symbiotic patterning events remains limited, and this remains a key mechanistic gap. The major CK transport routes and their implications for cytokinin partitioning in developmental and symbiotic contexts are summarized in Figure 2.

## 4. Integrated Signaling: Core Crosstalk Between Cytokinin and Other Plant Hormones

Plant growth, development, and environmental responses are orchestrated by a complex network of hormonal signals rather than by linear actions of individual hormones. Cytokinin resides at a central node within this network, and its functions are realized through synergistic or antagonistic crosstalk with other hormones.

Interaction with Auxin: This represents one of the best known and most pivotal interaction in plant hormone research. During the development of both roots and shoots, the two hormones typically exhibit an antagonistic relationship [42]. In the root apical meristem, cytokinin promotes cell differentiation by upregulating the expression of *SHY2/IAA3*. This, in turn, suppresses auxin signaling and transport, primarily by inhibiting the expression of *PIN* genes; Conversely, auxin promotes cell division and maintains meristem size by triggering the degradation of *SHY2/IAA3* proteins, thereby relieving this inhibition [42]. In callus tissue, a high cytokinin-to-auxin ratio induces shoot formation, whereas a low ratio promotes root formation. This principle serves as the cornerstone of plant tissue culture [3]. However, under certain contexts, such as in the maintenance of the shoot apical meristem, the two hormones can also act synergistically [43].

Interaction with Ethylene: Cytokinin induces ethylene biosynthesis primarily by upregulating the gene expression and modulating the protein stability of two key enzymes in the ethylene synthesis pathway: ACC Synthase (ACS) and ACC Oxidase (ACO) [37]. This interaction plays a critical role in regulating root growth and, as will be discussed in detail later, in root nodule symbiosis.

Interactions with Abscisic Acid (ABA) and Other Hormones: The interaction between cytokinin and the stress hormone ABA is particularly critical when plants respond to abiotic stresses, such as drought and salinity. Typically, stress triggers a rise in ABA levels alongside a decline in cytokinin concentrations within the plant; these coordinated changes collectively mediate adaptive responses [44]. Furthermore, cytokinin exhibits extensive signaling crosstalk with Gibberellins, Jasmonates, Salicylic acid, and others. Together, they form a sophisticated regulatory network that enables the plant to mount integrated responses to complex internal and external environmental changes [45].

### 4.1. Cytokinin: A Central Coordinator of Plant Form and Function

Available evidence indicates that CK functions as an important coordinator of plant growth, development, and environmental responsiveness. Its effects are rarely autonomous; rather, CK acts through interactions with auxin, ethylene, ABA, sugars, and nutrient signals to modulate developmental competence in a context-dependent manner [43,44,45].

### 4.2. The Auxin–Cytokinin Balance Is a Central Principle Governing Plant Morphogenesis

Shoot and root apical meristems: In the shoot apical meristem (SAM) and root apical meristem (RAM), antagonistic interactions between cytokinin and auxin maintain stem cell homeostasis. Cytokinin promotes differentiation in the RAM by inducing *SHY2/IAA3*, which suppresses auxin transport, while auxin maintains meristem activity by promoting *SHY2/IAA3* degradation. This mutual antagonism underpins the classic tissue culture principle: high cytokinin/auxin ratios promote shoot regeneration, while low ratios favor root formation [2].

Apical dominance: Auxin from the apical meristem suppresses lateral bud outgrowth via basipetal transport. Cytokinin synthesized in roots and transported shootward antagonizes this inhibition, promoting bud release. The dynamic balance between these hormones determines branching architecture and light capture capacity [1].

Leaf senescence: Cytokinin delays senescence—a phenomenon known as the Richmond–Lang effect—by maintaining chlorophyll and protein synthesis and promoting nutrient mobilization to leaves [1]. This has been harnessed biotechnologically: expressing IPT under the senescence-specific SAG12 promoter delays senescence and can increase biomass or yield in multiple species when promoter context and environment are favorable [46,47,48].

## 5. The Functional Landscape of Cytokinin: Orchestrating Plant Physiological Processes

### 5.1. Deciphering Cytokinin Heterogeneity at Single-Cell Resolution

Traditional hormone studies, which rely on bulk measurements from whole organs or mixed tissues, often obscure the cellular heterogeneity of CK responses. Single-cell and spatial transcriptomic approaches are beginning to resolve this complexity by identifying which cells become CK responsive, when they do so, and how those responses are coupled to neighboring hormonal and developmental states.

ScRNA-seq studies have enabled the mapping of transcriptomes across different cell types in the *Arabidopsis* root and have identified cell type-specific marker genes [49]. These studies have revealed that the response to cytokinin can exhibit significant heterogeneity even between adjacent cells.

For example, during the regeneration of shoots from callus, single-cell transcriptomic analysis has revealed that, despite the external application of high concentrations of cytokinin, only specific cell clusters (such as those exhibiting quiescent center-like identity) activate key stem cell regulators like *WUSCHEL* (*WUS*), thereby initiating the shoot meristem program [50]. This indicates that a cell’s intrinsic state and identity, rather than merely the hormone concentration, determine its competence to respond to hormonal signals.

Spatial transcriptomics takes this a step further by resolving gene expression profiles while preserving the spatial information of the tissue [51]. In a study of adventitious root regeneration in poplar, spatial transcriptomics revealed that the activation of cytokinin-responsive genes during root primordium formation is highly spatially specific. Furthermore, the promoters of these genes are enriched for auxin response elements (AREs), providing direct evidence for the synergistic interaction of the two hormones at precise spatial locations [51]. These emerging technologies are reshaping our understanding of hormone regulation by revealing an unexpected degree of complexity and precision in CK responses at single-cell and spatially resolved scales.

### 5.2. Mediating Environmental Responses: The Dual Role of Cytokinin in Abiotic Stress Tolerance

Negative Regulatory Role: Under stress conditions such as drought and high salinity, cytokinin typically acts as a negative regulator of root growth [44]. Stress inhibits cytokinin biosynthesis, and high levels of cytokinin can exacerbate the inhibitory effect of stress on root growth [52]. The impact of altered cytokinin metabolism extends beyond roots to photosynthetic tissues; studies in transgenic tobacco have shown that modifying cytokinin metabolism affects not only cytokinin but also auxin and abscisic acid contents in leaves and chloroplasts, and alters chloroplast ultrastructure [53]. Therefore, reducing endogenous cytokinin levels by overexpressing *CKX* genes can promote root growth, leading to a more extensive root system, thereby enhancing plant tolerance to drought and salt stress. This strategy has shown substantial translational potential in various crops [37].

Protective Function: On the other hand, cytokinin also has direct protective effects. It can regulate stomatal opening and closure, protect the photosynthetic apparatus from damage, and induce the synthesis of some protective substances [54]. For example, exogenous application of cytokinin can induce anthocyanin biosynthesis, and anthocyanins are important antioxidants with photoprotective functions [55].

Integration with Stress Hormones: These opposing roles are integrated through crosstalk with ABA and ethylene. Stress-induced ABA accumulation suppresses cytokinin signaling, while cytokinin modulates stress-responsive pathways to balance growth and survival [44].

### 5.3. Cytokinin in Plant–Microbe Symbiosis

The developmental and physiological modules reviewed above provide the necessary backdrop for interpreting the symbiosis literature, because symbiotic cytokinin signaling is more usefully understood as redeployment of pre-existing regulatory modules rather than invention of entirely new CK logic. In plant–microbe associations, CK-related outputs appear to arise from the reconfiguration of established modules for synthesis, transport, perception, and feedback control in new cellular and ecological settings [36,56,57]. The practical question is therefore not whether cytokinin is uniformly promotive or inhibitory, but which CK pool, in which tissue, at which developmental stage, and under which nutrient regime, is being altered.

Taken together, the current literature supports an important asymmetry between the two major symbiotic contexts discussed here. In arbuscular mycorrhiza, CK-associated outcomes remain heterogeneous and are most usefully interpreted through an evidence matrix that separates host genotype, fungal partner, nutrient regime, timing, and readout class [44,58,59]. In legume–rhizobium symbiosis, by contrast, the mechanistic case is considerably stronger: CK is more directly linked to cortical developmental reprogramming, coordination with infection-associated signaling, and systemic control of nodulation [42,60,61,62,63]. This asymmetry provides a useful framework for the following sections and helps avoid over-generalization across symbioses.

### 5.4. Cytokinin in Arbuscular Mycorrhizal Symbiosis: Context-Dependent Regulation

In arbuscular mycorrhizal (AM) symbiosis, CK effects are best interpreted as conditional outputs of a context-sensitive regulatory system rather than as a single uniformly positive or negative pathway. Outcomes vary with host genotype, fungal partner, phosphate or nitrogen status, developmental stage, tissue layer, and experimental readout [23,64]. By contrast, in legume nodulation the evidence for a direct CK requirement in cortical organogenesis is substantially stronger. We therefore treat the AM literature primarily as a structured evidence matrix and ask which biological layer a given experiment is actually interrogating, rather than forcing heterogeneous results into a simple positive-versus-negative CK model [44,65,66].

AM symbiosis is an ancient mutualism in which fungi enhance mineral nutrient uptake, especially phosphate, in exchange for plant-derived carbon. Compared with nodulation, however, CK functions in AM appear less tightly coupled to a discrete de novo organogenic program and more strongly filtered through physiological and environmental context. Many AM studies measure colonization, arbuscule abundance, nutrient transfer, or net host benefit, but these outputs are not interchangeable [44,58,59,67]. This asymmetry is important evolutionarily: AM largely modifies accommodation within pre-existing root tissues, whereas legume nodulation requires the activation of a specialized cortical developmental program. A plausible comparative interpretation is therefore that CK remained embedded in a broader nutritional and developmental network in AM, but was more strongly amplified and redeployed during the evolution of nodulation, where cortical cell cycle re-entry and organogenesis became central symbiotic outputs [42,56,59].

A practical synthesis is to distinguish two regulatory layers and to state explicitly which layer a given experiment is most likely to perturb. The first is local root competence, in which CK can influence epidermal entry, cortical accommodation, and arbuscule differentiation through effects on cell identity, sink strength, and root developmental state, especially under low phosphate and within defined developmental windows [23,59]. The second is whole-plant resource coupling, in which CK-associated changes may report broader shifts in nutrient sufficiency, carbon allocation, or host benefit thresholds rather than a direct local effect on fungal compatibility. At present, support is stronger for the first layer, whereas the second should still be treated as a provisional interpretive model rather than as a settled mechanism [44,57,59,60].

This two-layer framework also permits a more explicit working model for the apparent bidirectionality of CK effects in AM. Under nutrient-poor conditions, or when CK elevation is spatially restricted to responsive root domains, CK may lower the effective threshold for cortical accommodation and help sustain the local sink activity required for arbuscule development. Under more nutrient-sufficient conditions, or when CK elevation is broader and more systemic, the same signaling network may instead shift host outputs away from arbuscule maintenance and carbon allocation. We emphasize that this is a testable working model rather than a closed mechanism. Direct causal evidence linking broader or more systemic CK elevation to host cost–benefit evaluation in AM remains limited. The value of this framework is therefore primarily predictive: it suggests that CK chemistry, perturbation route, spatial scale, and developmental timing should differentially affect fungal entry, arbuscule persistence, nutrient transfer, and host performance [11,44,59,67].

To make this logic explicit and to enable meaningful comparison across studies, we organize the AM literature into an evidence matrix (Table 2) that classifies each study by CK chemistry, route of perturbation, spatial scale, symbiotic output class, and the relevant biological context, including host–fungus pairing, nutrient regime, and developmental stage [4,59]. Under such a framework, apparently contradictory outcomes need not be mutually exclusive, because different studies may perturb different CK pools, act at different spatial scales, or quantify different phases of the symbiosis rather than the same biological process under identical conditions [44,57].

Temporal sampling adds a further layer of interpretive risk. CK perturbations applied before fungal entry, during arbuscule differentiation, or after the symbiosis has become nutritionally productive do not interrogate the same biological process [44]. Future AM studies should therefore align perturbation timing with developmental staging and should pair colonization metrics with arbuscule status, nutrient transfer, and host carbon economics; otherwise, permissive and inhibitory CK effects can be conflated under a single and overly coarse label of “mycorrhizal outcome” [59].

This interpretation should be viewed as a provisional organizing framework rather than a settled mechanism. Even so, it generates testable predictions: (1) distinct CK species and transport routes should have non-equivalent effects on fungal entry versus arbuscule maintenance; (2) low-Pi, carbon-limited, and high-Pi conditions should differentially reshape CK output classes; and (3) cell type-resolved reporters should reveal whether AM-responsive CK signaling is concentrated in specific epidermal, cortical, or arbuscule-containing cell populations. Evidence status summary for AM: direct causal support is strongest for context dependence itself, moderate for local CK effects on accommodation-related outputs, and still limited for broad systemic resource budget interpretations.

A key practical implication is that negative AM phenotypes after systemic cytokinin elevation should not automatically be interpreted as evidence for a direct anti-mycorrhizal role of cytokinin. In many cases, a more conservative interpretation is that elevated cytokinin signals relative nutrient sufficiency or altered carbon economics, thereby reducing host willingness to maintain fungal investment. Dissecting these alternatives will require experiments that measure fungal structures, nutrient fluxes, and host carbon allocation in parallel [44,58].

## 6. Cytokinin in Legume–Rhizobium Symbiosis

Compared with AM symbiosis, where CK outputs are strongly shaped by host and environmental context, legume–rhizobium symbiosis provides much stronger evidence for a direct organogenic role of CK. In legumes, CK is not merely permissive; it is a core regulatory input into cortical cell cycle re-entry, primordium formation, and the coordination of infection with organ development.

### 6.1. Nodule Organogenesis: The Unique Cortical Response to Cytokinin in Legumes

Current evidence supports the view that a central event in root nodule symbiosis is the reactivation of cell division in cortical root cells, giving rise to the nodule primordium [42,60,61]. A defining feature of legumes is that cortical cells acquire strong developmental responsiveness to CK, allowing this hormone to function as an organogenic cue rather than only as a general growth regulator [42,60,61,68].

### 6.2. Working Model: Quantitative Gating Hypotheses and Their Current Limits

Recent live-imaging work has revealed periodic (~6 h) CK responses during rhizobial infection and nodule development, adding a temporal dimension to infection site selection and nodule positioning [69]. These findings suggest that CK responsiveness is not continuously permissive along the root but instead fluctuates over time and space, thereby helping define competent windows for symbiotic entry and organ initiation [69]. At present, the existence of this rhythmicity is better supported than any specific downstream coupling mechanism, and its quantitative relationship to auxin transport, infection-thread progression, and primordium spacing remains to be resolved.

This temporal behavior represents an important conceptual advance in nodule biology, but its underlying mechanism remains unresolved. It is therefore more accurate to regard rhythmic CK signaling as an emerging layer of nodulation competence rather than as a fully established cell-autonomous oscillator.

### 6.3. Established Versus Inferential Nodes in the Nodulation Cytokinin Network

It is useful to distinguish a genetically anchored core module from more inferential quantitative interpretations in the nodulation cytokinin network. Available evidence most strongly supports a core set of established nodes, including Nod factor-triggered CK accumulation and response, receptor requirements such as CRE1/LHK1 [60,61], induction of cortical cell division, and recruitment of downstream transcriptional regulators including NIN-linked programs [70,71,72]. By contrast, ideas such as transporter-defined local maxima, epidermis-versus-cortex threshold asymmetry, oscillatory competence windows, or an infection-suppressive ceiling are better regarded as working models that organize current observations but are not yet equally resolved in causal terms [68,69,71].

Within this evidence structure, the unusually strong cortical responsiveness to CK observed in nodulating legumes is best interpreted not as a generic property of all plant roots, but as a lineage-specific elaboration of pre-existing CK developmental circuitry [42,56]. This point matters mechanistically as well as evolutionarily. Unlike AM symbiosis, which primarily remodels accommodation within existing root tissues, nodulation requires de novo cortical organogenesis, sustained cell cycle re-entry, and coordinated patterning of a new symbiotic organ. These demands provide a clear biological rationale for why CK-dependent developmental machinery could have been selectively amplified and redeployed during legume evolution. The strongest genetic anchor for this interpretation remains receptor-level evidence: in Medicago truncatula, cortical CK responsiveness depends on CRE1; in Lotus japonicus, it depends on the homologous receptor LHK1; and a dominant gain-of-function LHK1 allele can trigger spontaneous nodules even in the absence of rhizobia [42,61].

Additional regulatory nodes further indicate that the nodulation CK network is not a simple linear growth-promoting pathway, but a constrained developmental program layered with immune and resource-sensitive feedback. In addition to core components such as NIN and Type-B RRs [42,72], recent work has identified regulators such as the pathogenesis-related protein PRP1, which is directly activated by NIN and negatively regulates root nodule symbiosis in *Lotus japonicus* [73]. Such nodes strengthen the interpretation that CK-driven organogenesis in legumes is embedded within a broader network that calibrates symbiotic progression, immune restraint, and investment cost, rather than operating as an isolated developmental switch.

### 6.4. Molecular Mechanisms of Nodule Initiation

Nodule initiation is orchestrated by multiple interconnected signaling pathways, with CK at the center of a broader regulatory network rather than acting in isolation.

Upstream signals: Rhizobial signal molecules, namely Nod factors (NFs), are recognized by receptors on the root hair cell surface, triggering a series of downstream signaling events, among which rhythmic intracellular calcium oscillations (calcium spiking) are central. This calcium signal is decoded by a calcium/calmodulin-dependent protein kinase (CCaMK) [74].

Nutrient context also enters this pathway very early. In Lotus japonicus, nitrate suppresses the rhizobia-induced upregulation of cytokinin biosynthesis and activation genes in the susceptible zone, thereby attenuating the cytokinin trigger for organogenesis and increasing nitrate sensitivity in cytokinin-deficient backgrounds [70]. These results indicate that nodulation-associated cytokinin signaling is shaped not only by rhizobial cues but also by the plant nutritional context [36,70]. Overall, current evidence strongly supports the organogenic role of CK in nodulation [42,60,61], whereas quantitative interpretations involving temporal patterning and related spatial models are better regarded as working models that remain only partly resolved [68,69].

This nitrate-sensitive CK node is especially important conceptually because it links environmental nitrogen status to the transient symbiotic CK burst in the susceptible root zone. In other words, nitrate does not merely reduce nodulation “downstream”; it can attenuate nodulation by dampening the local cytokinin biosynthetic and activation program that normally licenses cortical organogenesis [70].

Type-B RRs as downstream effectors: CK signaling converges on Type-B RRs. In *Medicago truncatula*, *MtRRB3* is required for normal nodulation and links receptor activation to transcriptional programs that promote cortical division and primordium development [71,72].

NIN as a central hub: NODULE INCEPTION (NIN) functions as a central regulator coordinating infection and organogenesis [42,72]. The CK-NIN relationship is one of the strongest mechanistic links in legume symbiosis, although the precise architecture of feedback between receptors, Type-B RRs, and NIN likely varies among species and developmental stages.

#### 6.4.1. Hormonal Interaction I: The Cytokinin–Flavonoid–PIN Axis and Local Auxin Maxima in the Nodule Primordium

As with the initiation of other plant organs, nodule formation requires a local auxin maximum at the incipient primordium [2].

In *Medicago truncatula*, current evidence supports a model in which CK signaling acts upstream of auxin redistribution, at least in part through flavonoid-associated regulation of PIN-dependent auxin transport, thereby contributing to local auxin accumulation in the developing primordium [42,75]. However, the extent to which this process also depends on additional auxin transporters, auxin metabolism, and cell type-specific feedback regulation remains unresolved [42,76].

**Figure 3:**
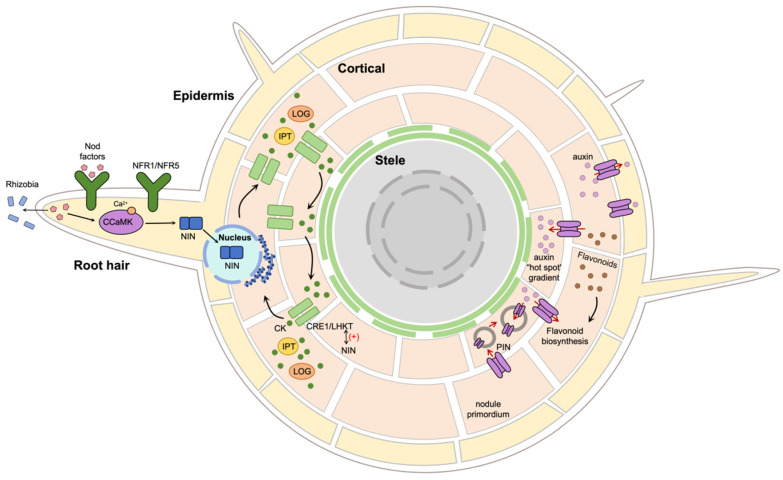
A spatial threshold framework for cytokinin action in legume–rhizobium symbiosis, integrating epidermal infection with cortical organogenesis. The model proposes that distinct cell layers interpret CK signals using different thresholds, competence states, and feedback constraints, thereby allowing infection and nodule organogenesis to be coordinated without requiring identical CK outputs in all tissues. Established genetic nodes are distinguished conceptually from quantitative gating features that remain inferential.

In *Medicago truncatula*, inhibition of flavonoid synthesis blocks the rhizobia-induced reduction in auxin transport and suppresses nodule formation [75]. In Glycine max, CK signaling can remodel the polar distribution of the key auxin transporter GmPIN1b in primordium cells [76]. Taken together, these findings support a mechanistic chain in which CK signaling contributes to flavonoid/PIN-mediated auxin redistribution, thereby helping establish the auxin maximum required for cortical organogenesis. Figure 3 integrates these ideas into a spatial threshold model linking epidermal infection with cortical organogenesis.

#### 6.4.2. Hormonal Interaction II: Fine-Tuning Infection and Nodulation Through Ethylene and ABA

Negative regulation by ethylene: Ethylene generally acts as a negative regulator of nodulation and rhizobial infection [36]. Ethylene-insensitive mutants often display hypernodulation phenotypes, indicating that ethylene constrains both infection and organogenesis.

CK–ethylene link: CK signaling can promote ethylene biosynthesis [77]. In *Medicago truncatula*, Nod factor signaling induces local CK accumulation and signaling in the cortex, while ethylene provides a counterbalancing inhibitory input. Thus, the net nodulation outcome depends not on CK alone but on how CK is integrated with ethylene sensitivity, tissue identity, and developmental timing.

Coordinating Role of ABA: Abscisic acid (ABA) also acts as a negative regulator, capable of simultaneously suppressing Nod factor signaling in the root epidermis and cytokinin-induced cell division in the root cortex [78]. This allows the plant to integrate external stress signals (ABA is a key stress hormone) with the energy-costly nodulation process to make a unified decision.

Crucially, the recently identified periodic (~6 h) CK responses [69] add a temporal dimension to this spatial model. Rather than indicating a static permissive state, this rhythmicity may define recurring windows of cortical competence. A plausible, but still unproven, possibility is that periodic CK responsiveness interacts with dynamic auxin transport and local infection progression to improve spatial precision while reducing ectopic organogenesis. Direct causal links between CK periodicity and auxin flux remain a key target for future work.

### 6.5. Signal Supply: Local Biosynthesis, Transport, and Degradation During Nodule Development

To generate precise CK signals at the right time and place, biosynthesis, transport, and degradation are dynamically regulated during nodule development.

Biosynthesis: Nod factor treatment rapidly induces the expression of CK biosynthesis (IPT) and activation (LOG) genes in the root [36]. In *Lotus japonicus*, *LjIpt2* and *LjLog4* have been implicated in generating an early CK pulse associated with nodule initiation, and in *Medicago truncatula*, induction of *MtIPT3* supports local CK accumulation during cortical organogenesis [2,71]. Collectively, these studies support the view that local CK production contributes to the competence window for primordium formation.

Transport: The establishment of spatiotemporal CK gradients is likely crucial for nodulation. Reporter analyses indicate that CK response first appears in epidermal cells and then spreads to the cortex [36]. Transporters such as *MtABCG56* are plausible candidates for shaping these gradients, not simply by changing bulk CK abundance, but by redistributing effective CK pools between epidermal infection-competent domains and cortex-associated organogenic domains. Direct evidence linking defined CK species and in vivo transport fluxes to this epidermis–cortex balance is still limited, and the exact substrate specificity, transport direction under physiological conditions, and quantitative contribution of these transporters remain to be resolved.

Degradation: To prevent uncontrolled signaling, CK levels are tightly constrained by *CKX* genes. In *Lotus japonicus*, *LjCkx3* is induced in developing primordia, and perturbation of CKX function alters both infection and nodule number. Rather than supporting a simple “more CK is better” model, these results argue for an optimal CK window in which insufficient and excessive signaling are both detrimental, partly because elevated CK can reinforce inhibitory pathways such as ethylene signaling [68,79].

Evidence status summary for nodulation: Direct causal evidence is strongest for localized CK biosynthesis/signaling during cortical organogenesis [2,4,71] and for receptor-dependent activation of nodule developmental programs [42,60,61]. Support is growing, but still less definitive, for transporter-shaped gradients, oscillatory competence windows [69], and quantitatively defined infection ceilings [68]. For reference, the major cytokinin-related genetic nodes implicated in nodulation are organized in Table 3 according to species, functional role, network context, and supporting evidence.

### 6.6. Systemic Integration of Symbiosis with Whole-Plant Physiology

The role of CK in nodule symbiosis extends beyond local root events; it also participates in long-distance signaling that links nodulation with whole-plant physiology and nutritional status.

#### 6.6.1. Cytokinin as a Shoot-Derived Component of Autoregulation of Nodulation

Legumes have evolved a sophisticated autoregulation of nodulation (AON) system that uses long-distance signals to limit total nodule number, thereby balancing the benefits of nitrogen fixation against its carbon costs [80,81]. In the canonical model, successful root infection induces CLE peptides that move to the shoot through the xylem and are perceived by shoot receptors such as HAR1 in *Lotus japonicus*, SUNN in *Medicago truncatula*, and NARK in *Glycine max* [80,81]. Shoot perception is then followed by the generation of a shoot-derived inhibitory signal (SDI) that suppresses further nodulation in the root [80,82].

Current evidence therefore supports CK more strongly as a shoot-acting or systemic component within the inhibitory circuit than as the singular identity of the SDI itself. The most defensible interpretation is that CK functions within, or in parallel to, a multicomponent AON framework in which CLE peptides and their receptor modules define systemic state, while CK contributes to the amplitude, timing, or tissue sensitivity of the return inhibition imposed on the root. Nitrate status, carbon availability, and source–sink relationships are likely to modulate this same circuit, helping determine the final nodulation set point [36,70,83].

Operationally, this means that future AON studies should test CK in explicitly multicomponent designs rather than in isolation. Grafting, reciprocal transport assays, CK speciation, and tissue-resolved sensitivity measurements will be needed to determine whether CK behaves as a mobile inhibitory signal, a permissive amplifier of shoot-derived inhibition, or a context-setting hormone that alters how root tissues interpret the systemic return signal. Such a framework is more faithful to the available evidence than assigning CK sole pathway identity at present.

A related implication is that whole-plant nitrogen state, Nod factor signaling, and symbiosis competence likely reshape not only nodule number but also the broader root-associated ecological context. In Lotus japonicus, nitrogen and Nod factor signaling alter root exudate composition and the assembly of associated bacterial communities, consistent with the view that systemic symbiotic decisions are embedded in a wider host resource-allocation program rather than in an isolated nodule-counting module [84].

#### 6.6.2. Cytokinin as a Systemic Integrator of Carbon, Nitrogen, and Symbiotic Investment

This framework generates testable expectations. Perturbing transporter-defined CK gradients or localized CKX activity should shift the balance between infection and organogenesis, whereas manipulations that decouple CK flux from C/N status may increase nodulation but impose costs on vegetative growth or seed yield unless carbon supply is simultaneously enhanced. These expectations should be treated as design hypotheses pending direct causal tests across environments and genetic backgrounds.

Future work should combine grafting and reciprocal transport assays with isotope tracing (^13^C/^15^N), CK speciation, and cell type-resolved reporters to establish causal relationships between systemic CK dynamics, resource allocation, and symbiotic outcomes.

## 7. Translational Hypotheses and Synthetic Biology Perspectives

Understanding CK homeostasis has opened actionable routes for crop improvement, but the strongest successes have typically come from tuning CK signals in space and time rather than globally increasing CK. Across systems, the most transferable principle is to treat CK engineering as a constrained translational problem: choose a controllable variable (biosynthesis, degradation, transport, or perception), specify the target tissue and developmental window, and pair the intervention with assays that quantify both benefit and cost.

The cases in Table 4 therefore should be interpreted as translational exemplars rather than universal prescriptions. Robust deployment will require multi-environment validation, explicit reporting of promoter and tissue specificity, and systematic monitoring of tradeoffs (for example, lodging, altered root architecture, drought sensitivity, or immune costs) that may emerge when CK is shifted outside its native feedback range [85,86,87,88].

A practical editorial implication is that engineering sections in this field should increasingly report boundary conditions alongside successes: target tissue, promoter logic, nutrient regime, developmental stage, and measured costs in immunity, architecture, or resource allocation. Without these descriptors, apparent “success stories” are difficult to generalize across crop backgrounds or environments [86,87,88].

## 8. Conclusions

### 8.1. Conclusions and Future Directions

Comparative, genetic, and physiological evidence supports a view of cytokinin (CK) as an evolutionarily repurposed signaling system that links development with resource status and, in some lineages, with symbiotic investment. The strongest mechanistic support concerns legume nodulation, where CK is integrated into a specialized cortical developmental program. By contrast, evidence from arbuscular mycorrhiza (AM) still supports a more context-dependent picture, with CK effects varying according to host background, fungal partner, nutrient conditions, developmental timing, and experimental readout.

Taken together, the literature is most consistently organized by three recurring interpretive dimensions: compartmentalized CK pools, context-dependent decoding of effective CK signals, and the coupling of local developmental responses to whole-plant nutritional status. We use these dimensions as a comparative framework rather than as a single closed explanatory model, because they are not equally resolved in every biological context. They help explain why bulk CK measurements are often insufficient predictors of biological output, while also highlighting where mechanistic resolution remains weakest, including transporter-resolved CK partitioning and systemic integration.

Future progress will depend on moving from descriptive hormone landscapes to causal, cell-resolved models. The immediate priority is to determine which CK species are produced, transported, perceived, and degraded in defined cell types during specific developmental windows. This will require matched analyses of CK speciation, tissue-restricted genetics, transporter perturbation, receptor function, and quantitative reporter outputs.

The field also now has an opportunity to integrate classic hormone genetics with single-cell, spatial, and quantitative imaging approaches. Such integration should clarify how local CK responses are coordinated with broader nutrient-status signals, how nutrient context reshapes symbiotic competence, and which parts of the network are robust enough to support translational design. In this sense, the next phase of cytokinin research is not only to catalog additional components, but to build predictive frameworks that explain when and where CK signaling changes plant developmental and symbiotic decisions.

Recent receptor-rewiring studies also suggest that developmental output specificity can, in principle, be engineered with far greater precision than previously assumed. Integrating such receptor-level design with CK speciation, transporter genetics, and spatially resolved response mapping should allow the field to move from descriptive hormone landscapes toward more predictive control of symbiotic developmental decisions, but this prospect still awaits robust validation beyond a limited number of systems [92].

### 8.2. Priority Directions for the Field

Priority 1. Cell type-resolved CK response functions in symbiosis. Combine CK reporters, single-cell/spatial transcriptomics, and cell type-specific receptor perturbation to derive dose–response relationships in the epidermis versus cortex (nodulation) and in arbuscule-containing cells (AM).

Priority 2. Transport route causality. Map and perturb CK transporters and metabolic enzymes in defined tissues to distinguish local effective CK pools from systemic CK streams, and test whether proposed gradients and thresholds are transport-dependent.

Priority 3. Systemic integration and trade-offs. Quantify how nutrient status (especially phosphate and nitrogen) reshapes CK pools and CK sensitivity across organs, and determine how these systemic signals gate symbiotic investment via AON and carbon/nitrogen budgeting.

Together, these priorities argue for a next-generation cytokinin field that is less centered on bulk hormone abundance and more centered on effective signaling landscapes defined by cell type, transport route, developmental timing, and resource context.

## Figures and Tables

**Table 1:** Cytokinin Signaling Evolution: Key Adaptations and Evidence Evaluation.

Evolutionary Innovation Event.	Key Genes/Components	Functional Significance	Comparative Evolutionary Interpretation	Supporting Literature
Emergence of the core TCS module	Core histidine kinases (HKs) and response regulators (RRs)	Establishes a His–Asp phosphorelay signaling module	Provides a reusable ‘integrative layer’ for coupling environmental sensing to developmental regulation, later specialized for hormone control	[9]
Emergence of CHASE- containing HK receptors	CHASE-HK receptor	Confers cytokinin-specific perception via CHASE domain receptors	Links an ancestral phosphorelay to hormone-defined inputs, supporting context-specific developmental and symbiotic deployment	[27]
The emergence of *AP-IPTs*	Adenosine phosphate IPTs (*AP-IPTs*)	Activates efficient de novo synthesis of highly active CKs (*t*Z, iP)	Enables expansion of bioactive CK pools and tighter coupling of nutrient status to multicellular developmental programs	[28]
Differentiation of Type-A and Type-B RRs	Type-A and Type-B RRs	Establishes a positive activation and negative feedback loop	Ensures the transience, robustness, and precise control of signals	[7]
Expansion of the *CKX* gene family	*CKX* gene	Enables spatiotemporally specific degradation of CKs	Fine-tuned regulation of CK homeostasis, balancing growth and stress responses	[17]

**Table 2:** Evidence matrix for context-dependent regulation of AM symbiosis by cytokinin.

Host	Fungus	Nutrient Context (P/N)	CK Chemistry	Route of Perturbation	Spatial Scale	Symbiotic Output Class	Outcome Direction	Primary Ref(s)/Inference Level
*Pisum sativum (pea)*	*Glomus intraradices*	Low Pi; early growth	BAP	Exogenous application	Root-local	Accommodation (arbuscule abundance)	Negative (reduced arbuscule incidence)	[59]
*Medicago truncatula*	*Gigaspora margarita*	Low Pi; colonization stage	Endogenous CK signaling (not chemically resolved)	Receptor mutant, cre1 (*MtCRE1*)	Root-local	Colonization, arbuscules, and host growth	Null/conditional (colonization largely maintained; root architecture altered)	[57]
*Medicago truncatula*	*Rhizophagus irregularis*	Low Pi; early colonization	Exogenous CK	Exogenous application	Root-local	Entry versus accommodation (separable readouts)	Context-dependent	[57,59]
*Nicotiana tabacum (tobacco)*	*Rhizophagus irregularis*	Moderate Pi	Reduced root CK	*CKX* overexpression	Root-local/systemic effect unresolved	Colonization rate and accommodation	Negative (decreased colonization)	[58]
*Nicotiana tabacum (tobacco)*	*Rhizophagus irregularis*	Moderate Pi	Increased root CK	*IPT* overexpression	Root-local/systemic effect unresolved	Colonization rate and early accommodation	Positive in early stages	[58]
*Various hosts*	*Various AM fungi*	High Pi/low nutrient demand	Not chemically resolved	Conceptual synthesis (not primary experimental perturbation)	Systemic/whole-plant	Carbon allocation and colonization tendency	Often negative/conditional; consistent with nutrient-sufficiency signaling	[23,36]

**Table 3:** Genetic functions and developmental context of cytokinin-related genes in nodulation.

Gene/Family	Species	Evidence-Anchored Role in Nodulation	Network Context	Key Phenotype/Evidence	Primary/Supporting Ref(s)
*CRE1/LHK1*	*M. truncatula/L. japonicus*	CK perception; initiates cortical cell division and nodule organogenesis	Upstream of NIN; intersects with ethylene/ABA constraints	Loss-of-function reduces nodulation and primordium development; gain-of-function LHK1 can trigger spontaneous nodules	[60,61,68]
*Type-B RR module*	*M. truncatula*	Transcriptional activation downstream of CK perception during nodule initiation	Links CK signaling to cell cycle and organogenic transcriptional programs	Reduced nodule number	[71,72]
*NIN*	*M. truncatula/L. japonicus*	Master regulator coordinating infection and organogenesis	Forms positive feedback with CRE1; activates CLE peptide genes in the AON module	Severely impaired or absent nodulation	[42,72]
*NSP1/NSP2*	*M. truncatula/L. japonicus*	Core Nod factor signaling components required for nodulation	Functionally intersect with CK-triggered organogenic programs	Nodulation defective	[72,78]
*MtIPT3*	*M. truncatula*	CK biosynthesis during nodule development	Supports local CK accumulation and downstream responses	Reduced nodule number and altered nodule development when perturbed	[2,71]
*LjIpt2/LjLog4*	*L. japonicus*	Candidate CK biosynthesis/activation nodes during nodule initiation	Likely contribute to local CK supply; exact hierarchy remains context dependent	Reduced initiation efficiency reported in specific genetic backgrounds	[2,36]
*LjCKX3*	*L. japonicus*	CK degradation and homeostatic buffering during root and nodule development	Buffers CK levels during root and nodule development	Reduced nodulation and altered infection when perturbed	[79]
*MtABCG56*	*M. truncatula*	Candidate CK transport/distribution component during nodulation	May contribute to epidermis–cortex signal partitioning	Perturbation is associated with nodulation defects, but mechanistic resolution remains incomplete	[36]

**Table 4:** Design variables, genetic targets, constraints, and validation assays for cytokinin pathway engineering.

Design Variable	Genetic Lever	Target Crop/Context	Expected Gain (Phenotype)	Major Constraints/Trade-Offs	Required Validation Assays	Key Ref(s)
Increase Yield	*OsCKX2* (RNAi/ CRISPR)	Rice	Tiller number ↑, Grains per panicle ↑, 1000-grain weight ↑	Promising for yield, but requires stage- and tissue-specific tuning; monitor growth–stress trade-offs.	Multi-site field trials; yield components; CK profiling; stress trade-off tests	[89]
Delay senescence	*SAG12::IPT*	Tobacco	Delayed leaf senescence, Biomass ↑	Can delay senescence and improve biomass, but developmental timing and source–sink penalties must be checked.	Multi-site field trials	[46]
Context-specific trait optimization	*AtMYB32xs::IPT*	Canola	Delayed leaf senescence, Drought tolerance ↑	Drought-associated gains depend on promoter behavior and environment; monitor pleiotropic effects.	Field trials; drought physiology; root imaging	[47]
Stress tolerance	*W31::* *CaCKX6* (Root- specific)	Chickpea	Root growth ↑, Drought tolerance ↑	May enhance drought tolerance with limited nodulation penalty; co-evaluate root architecture and N fixation.	Field trials; drought physiology; root imaging; nodulation/N-fixation assays	[90]
Context-specific trait optimization	*OsCKX2* (CRISPR)	Rice	Water retention capacity ↑, Photosynthetic function ↑	Improved drought survival is promising, but effects on architecture and reproduction need validation.	Field trials; drought physiology; root imaging; recovery assays	[21]
Context-specific trait optimization	*PSARK::* *IPT*	Peanut	Delayed leaf senescence, Drought tolerance ↑	Reported drought-associated yield gains require validation across soils and seasons.	Field trials; drought physiology; root imaging; recovery assays	[91]
Context-specific trait optimization	*AtMYB32xs::IPT*	Wheat	Delayed leaf senescence, Drought tolerance ↑	Water-stress gains are encouraging, but promoter behavior, phenology, and yield stability remain constraints.	Field trials; drought physiology; root imaging; recovery assays	[48]

Note: ↑ indicates an increase, enhancement, or improvement in the measured parameter or trait compared to the control (e.g., “Tiller number ↑” means an increase in tiller number).

## Data Availability

All data discussed are available in the cited references. No new data were generated for this study.

## Abbreviations

CK, cytokinin; AM, arbuscular mycorrhiza; AON, autoregulation of nodulation; NF, Nod factor; RR, response regulator; HK, histidine kinase; HPt, histidine phosphotransfer protein; MSP, multi-step phosphorelay; TCS, two-component system; SAM, shoot apical meristem; RAM, root apical meristem; IPT, isopentenyltransferase; LOG, LONELY GUY; CKX, cytokinin oxidase/dehydrogenase; CLE, CLAVATA3/EMBRYO SURROUNDING REGION-related peptide; SDI, shoot-derived inhibitory signal; NIN, NODULE INCEPTION.
